# Oxygen Vacancy–Redox Synergy in a Zr/Fe Metal–Organic Framework Enables Highly Selective Uranyl Photoreduction and Immobilization

**DOI:** 10.34133/research.1425

**Published:** 2026-09-09

**Authors:** Taohong Xu, Lijuan Feng, Peng Liu, Xinhang Zhao, Hui Wang, Guanbing Zhou, Ning Wang

**Affiliations:** ^1^ School of Marine Sciences, State Key Laboratory of Marine Resource Utilization in South China Sea, Hainan University, Haikou 570228, China.; ^2^ State Key Laboratory of Tropic Ocean Engineering Materials and Materials Evaluation, Hainan University, Haikou 570228, China.

## Abstract

Addressing uranium contamination in tailings wastewater is key to reducing ecological threats and securing the future of nuclear power. The integration of adsorption with in situ photocatalytic U(VI) immobilization has been explored as a green alternative, but charge separation inefficiency and poor ion selectivity continue to hinder its use in complex wastewater. Herein, we constructed a bimetallic metal–organic framework, OVs-Zr/Fe UiO-66-NH_2_, by incorporating Fe^2+^/Fe^3+^ species into the UiO-66-NH_2_ lattice. The incorporation of Fe into the UiO-66-NH_2_ lattice narrows the bandgap and improves visible-light absorption. The same Fe doping induces oxygen vacancies (OVs) within the framework. The OVs trap photogenerated electrons to enhance charge separation, while their electron-rich nature favors the selective uptake of UO_2_^2+^. Meanwhile, the Fe^2+^/Fe^3+^ redox couple provides an internal electron mediation pathway, directing electrons to the OV-bound uranyl species and promoting the interfacial uranium conversion into stable species. With this combined adsorption–photocatalysis mechanism, OVs-Zr/Fe UiO-66-NH_2_ removed 99.2% of U(VI) within 6 h under ambient air without sacrificial agents, and its distribution coefficient (*K*_d_) reached 1.96 × 10^5^ ml g^−1^, 11.9 times that of pristine UiO-66-NH_2_. This work provides a synergistic strategy for crafting photocatalysts with high efficiency and selectivity for sustainable uranium remediation in complex waters.

## Introduction

Nuclear energy is becoming an important substitute for fossil fuels owing to its high energy density and low carbon footprint [1]. The main fuel for nuclear energy is uranium. It is an essential element in the energy-transition process. For decades, processes such as uranium mining and processing have generated a large amount of tailings [2]. These tailings continuously release leachate containing hexavalent uranium, which is not only chemically toxic but also radioactive, into the surrounding environment [3]. Treatment of tailings wastewater is necessary for the long-term sustainability of the nuclear energy industry as well as the well-being of the environment and local residents. There are a number of different methods that have been suggested such as adsorption [4,5], membrane separation [6,7], electrodeposition [8], ion exchange [9,10], and biological methods [11]. Among all, the adsorption method seems to be the most promising strategy because of its ease of operation, mild conditions, and effectivity at different concentrations of uranium [12,13]. Despite the above, there are clear limitations with traditional adsorbent materials. Limited availability of active sites, relatively low rate of adsorption process as well as the presence of competing ions make the uranium removal process less effective [14]. It is quite clear that new more effective adsorbent materials should be developed.

Combining adsorption with photocatalytic approach increases the effectiveness of the uranium removal process. In this approach, photocatalytic immobilization turns soluble uranium into insoluble form. Then, the insoluble uranium ions leave the active adsorption site free for future adsorption process [15,16]. Such mechanism solves the problem of limited number of active sites and increases uranium extraction efficiency [17]. In fact, most of the existing photocatalytic systems show higher extraction capacity than alternative methods [18]. However, the vast majority of photocatalysts only respond to UV radiation, which contributes to about 5% of the whole solar radiation spectrum. To overcome the mentioned limitation, the development of visible-light active materials has been suggested as the way forward. Band structure engineering is a useful approach that may help to enhance the efficiency of solar energy utilization [19]. TiO_2_ [20], g-C_3_N_4_ [21], and CdS [22] are popular photocatalysts. However, these catalysts are not applicable in reduction of U(VI) due to fast recombination of electron–hole pairs. Sacrificial agents and inert atmosphere may alleviate the problem, but they are too expensive and need specific conditions. Consequently, development of photocatalysts that could effectively utilize visible light and avoid recombination remains one of the key issues.

There is a wide variety of competing cations such as Ba^2+^, Cd^2+^, Cu^2+^, Mg^2+^, Sr^2+^, and Co^2+^ in tailings wastewater. These ions may occupy the active centers of sorbent and compete with U(VI) for interaction with functional groups, thus lowering uranium selectivity [23]. Efficient U(VI) immobilization in a complex aqueous environment necessitates the design of highly selective and effective photocatalysts. Metal–organic frameworks (MOFs) have increasingly been gaining interest as potential platforms for photoreduction catalysis. The UiO-66-type MOFs possess unique properties, including extraordinary framework stability, adjustable pore size, and varied coordination environment [24,25]. Doping MOFs with metal ions not only lead to the modification of the electronic structure and the reduction of the bandgap for the harvesting of visible light but also results in the formation of active redox metal centers, which facilitate the creation of reversible electron transport pathways at the interfaces. Another approach includes the modification of the local structure of MOFs via introduction of oxygen vacancies, exposure of functional groups, or creation of coordinatively unsaturated metal centers. These structural defects not only assist in charge separation but also in providing appropriate coordination environment for uranium enrichment in some instances [26–28]. However, the way metal ion doping should be coupled with the control of local structure remains to be elucidated to improve U(VI) selectivity and immobilization efficiency simultaneously.

**Fig. 1.**
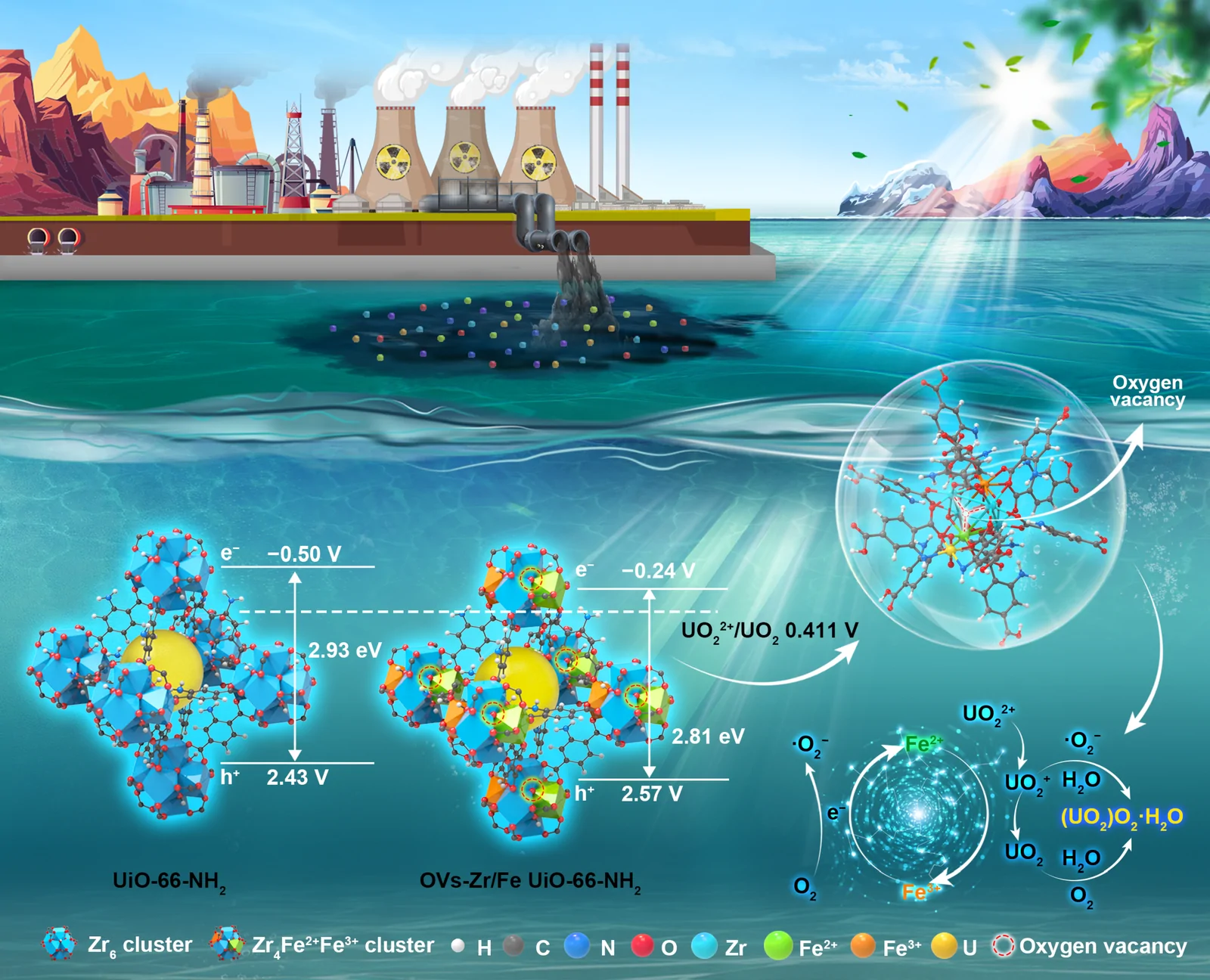
Reaction routes for the selective reduction of uranium over OVs-Zr/Fe UiO-66-NH_2_.

Based on the design strategy outlined above, we prepared a novel bimetallic OVs-Zr/Fe UiO-66-NH_2_ photocatalyst (Fig. 1). The incorporation of Fe initially tunes the electronic structure of UiO-66-NH_2_ through narrowing the band gap and increasing visible-light-absorption capacity. Moreover, it assists in creating oxygen vacancies (OVs) that serve as important interface centers responsible for regulating charge behaviors and capturing uranyl ions. Notably, the created OVs reduce charge recombination via scavenging photogenerated electrons, and their electron-rich local environment ensures that UO_2_^2+^ is selectively enriched relative to the others due to its higher affinity in complex environments. Furthermore, the redox cycle of the neighboring Fe^2+^/Fe^3+^ plays as an internal electron shuttle to promote electron transfer from OVs to uranyl ions for efficient photoreduction of U(VI). In addition, Fe-induced lattice distortion creates a hierarchical micropore/mesopore structure, which makes it easier for uranyl ions to diffuse through the material and reach the active sites. As a result, the OVs-Zr/Fe UiO-66-NH_2_ photocatalyst removes 99.2% of U(VI) within 6 h under ambient air with no sacrificial agents added, and its distribution coefficient (*K*_d_) reaches as high as 1.96 × 10^5^ ml g^−1^, 11.9 times the value for the pristine UiO-66-NH_2_. In this work, we have developed an integrated strategy that combines oxygen-vacancy-driven uranyl enrichment with Fe-based redox electron transfer, enabling selective uranium recovery via photocatalysis.

## Results and Discussion

### Structural and physicochemical characterization

**Fig. 2.**
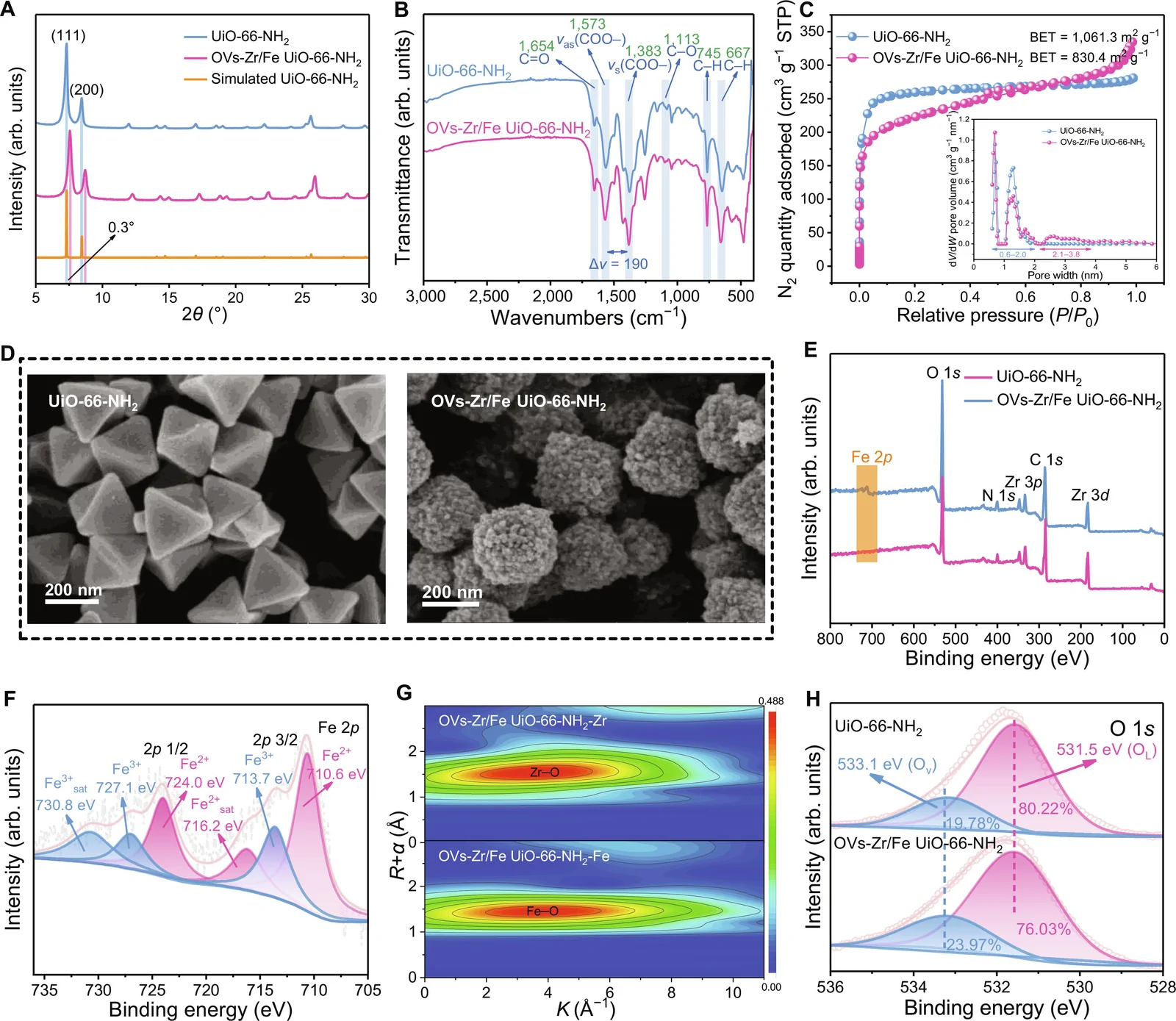
Characterizations of UiO-66-NH_2_ and OVs-Zr/Fe UiO-66-NH_2_. (A) X-ray diffraction (XRD). (B) Fourier-transform infrared (FT-IR) spectra. (C) N_2_ sorption isotherms and non-local density functional theory (NLDFT) pore-size distribution. STP, adsorbed volume reported at standard temperature and pressure; BET, surface area calculated by Brunauer–Emmett–Teller. (D) Scanning electron microscopy image. (E) X-ray photoelectron spectroscopy (XPS) spectra. (F) High-resolution Fe 2*p* XPS spectra. (G) Wavelet-transform extended x-ray absorption fine structure contour plots. (H) High-resolution O 1*s* XPS spectra.

The bimetallic MOF catalyst OVs-Zr/Fe UiO-66-NH_2_ was synthesized by tuning the Zr/Fe molar ratios during solvothermal synthesis. X-ray diffraction (XRD) patterns were collected for both OVs-Zr/Fe UiO-66-NH_2_ and the pristine UiO-66-NH_2_ (Fig. 2A). All characteristic diffraction peaks of the parent framework remain present in the modified material, confirming that its crystalline structure stays intact. Notably, the peak positions shift slightly, with 2*θ* values of the Fe-incorporated samples consistently rising by around 0.3°. It should be noted that while the overall XRD pattern confirms the preservation of the long-range framework topology, this slight peak shift indicates local lattice distortions at the metal nodes. This shift points to lattice distortion, likely caused by partial replacement of Zr^4+^ with Fe^2+^ and Fe^3+^ ions [29]. The difference in ionic radii (Zr^4+^ ≈ 0.84 Å, Fe^2+^ ≈ 0.92 Å, Fe^3+^ ≈ 0.785 Å) induces local lattice strain and further promotes the formation of oxygen vacancies [30]. Overall, the XRD results confirm that the framework keeps its crystallinity after metal incorporation. This is likely because of the low metal loading, high dispersion of Fe species, and the fact that Fe^2+^ and Fe^3+^ fit well into the coordination environment of the Zr-based nodes.

The chemical environment in the vicinity of the metal ions was characterized using Fourier-transform infrared (FT-IR) spectroscopy. As is seen in the FT-IR spectra of UiO-66-NH_2_ and OVs-Zr/Fe UiO-66-NH_2_ (Fig. 2B), typical absorptions due to the organic linkers were observed in both samples. Specifically, the C=O stretch vibration was detected at 1,654 cm^−1^, and the asymmetric and symmetric carboxylate vibrations were recorded at 1,573 and 1,383 cm^−1^, respectively. The frequency difference Δ*ν* (*ν*_as_(COO) − ν_s_(COO)) of 190 cm^−1^, characteristic of bridging coordination, proves bond formation between the metal centers and linkers [31]. Besides, both samples demonstrate additional characteristic bands: C–O stretch vibrations at 1,113 cm^−1^ and out-of-plane aromatic C–H out-of-plane bending vibrations at 745 and 667 cm^−1^ [32]. Overall, the IR spectra of OVs-Zr/Fe UiO-66-NH_2_ agree with those of the pristine compound, suggesting that the parent framework remains intact after the incorporation of iron. It can be rationalized by the fact that Zr^4+^ ions are substituted by highly dispersed Fe^2+^ and Fe^3+^ ions in an isomorphous manner, similarly coordinating with organic linkers as Zr^4+^ ions do, which corresponds to the results of XRD. Notably, the metal–oxygen (M–O) stretching vibrations are not distinctly visible in the FT-IR spectra due to the strong overlap with organic linker vibrations and the low Fe content, but the formation of metal–oxygen bonds is unambiguously corroborated by the complementary XRD and x-ray photoelectron spectroscopy (XPS) results.

Nitrogen adsorption–desorption experiments have been performed to determine the effects of metal incorporation on the porosity properties (Fig. 2C). The unmodified UiO-66-NH_2_ displays a Type I isotherm having H4 hysteresis loop. A steep increase in N_2_ adsorption under low relative pressure conditions (*P*/*P*_0_ < 0.1) has been detected which implies the formation of microporous structures [33]. In comparison, the OVs-Zr/Fe UiO-66-NH_2_ shows typical Type IV isotherm with an H1 hysteresis loop, which suggests that not only micropores but also ordered mesopores are present in this compound. Specifically, Fe doping leads to a decreased BET area (1,061.3 to 830.4 m^2^/g) and micropore volume (0.3899 to 0.2298 cm^3^/g), but a sharply increased mesopore volume (0.0446 to 0.2879 cm^3^/g), confirming the exclusive generation of mesopores (Table S1). To quantitatively analyze the pore structure, the original gas-adsorption data were reanalyzed using the NLDFT method (N_2_ at 77 K on carbon slit pores, non-negative regularization). The NLDFT pore-size distributions reveal that both samples exhibit microporous features corresponding to the intrinsic cage structure of the UiO-66 framework. Specifically, the UiO-66 framework contains tetrahedral and octahedral cavities of approximately 0.8 to 0.9 nm and 1.1 to 1.2 nm, respectively, connected through narrower triangular windows of approximately 0.6 nm. The pore-size distributions derived from gas adsorption are therefore discussed as effective cavity-size distributions. For OVs-Zr/Fe UiO-66-NH_2_, additional pore volume in the 2.1- to 3.8-nm range is observed, which may arise from interparticle voids or defect-induced pore enlargement rather than ordered framework mesopores. This secondary porosity, together with the intrinsic micropores, facilitates the diffusion of uranyl ions to the active sites (–NH_2_ and OVs) inside the porous network [34]. The generation of this secondary porosity is attributed to the heterovalent substitution of Zr^4+^ ions by Fe^2+^/Fe^3+^ ions, which induces local lattice strain and structural distortions.

Scanning electron microscopy and high-resolution transmission electron microscopy (HRTEM) images (Fig. 2D and Fig. S1) confirm that the pristine UiO-66-NH_2_ presents regular octahedral structures with clean surfaces and well-defined edges. For the OVs-Zr/Fe UiO-66-NH_2_ structure, the difference is evident since the original octahedra transform into irregular and aggregated spherical particles. Modification of the Fe^2+^/Fe^3+^ causes the change of crystal growth kinetics, affecting metal–ligand coordination and local nucleation. This change indicates that the new structure could expose more active sites which are easily accessible, and mass transfer would be better during the adsorption and photoreduction of U(VI) [35]. The energy-dispersive x-ray spectroscopy elemental mapping (Fig. S2) shows that the distributions of C, O, Zr, and Fe throughout the OVs-Zr/Fe UiO-66-NH_2_ framework are homogenous. The XPS spectra were recorded to determine the elemental states in the UiO-66-NH_2_ and OVs-Zr/Fe UiO-66-NH_2_. Survey spectra (Fig. 2E) confirm that the main elements are C, O, Zr, and Fe in accordance with the results from energy-dispersive x-ray spectroscopy elemental mapping. Peaks at the O 1*s* and Zr 3*d* peaks in UiO-66-NH_2_ suggest the existence of Zr–O bonds. In the same elemental regions, the Fe-modified sample is not different from the pristine one.

The Fe 2*p* signal of OVs-Zr/Fe UiO-66-NH_2_ is divided into 4 well-resolved peaks and 2 satellite peaks (Fig. 2F). Doublet peaks at 710.9 eV (Fe 2*p*_3/2_) and 724.1 eV (Fe 2*p*_1/2_) demonstrate spin–orbit coupling with a shift of 13.2 eV which agrees with the Fe^2+^ ion. Additional peaks at 713.6 and 727.1 eV are related to Fe^3+^ ion which is created through surface oxidation of metastable Fe^2+^ sites [36]. The heterovalent substitution of Zr^4+^ by Fe^2+^/Fe^3+^ induces oxygen vacancies to compensate for charge imbalance, while also generating local lattice strain and hierarchical porosity, confirming that Fe incorporation and vacancy generation are structurally coupled. The substitution of Zr by Fe was further confirmed by Zr K-edge and Fe K-edge extended x-ray absorption fine structure (EXAFS) analyses (Fig. 2G, Figs. S3 to S7, and Table S2). Well-resolved Fe–O and Zr–O coordination-shell signals are observed in wavelet-transform EXAFS contour plots (Fig. 2H). The high overlap of their peak positions in the *k*–*R* plane demonstrates that Fe sites adopt local coordination geometry analogous to Zr, confirming the successful incorporation of Fe into the MOF framework. The Zr–O coordination number decreases from 8.3 ± 0.8 to 7.7 ± 0.7 upon Fe incorporation, evidencing oxygen-vacancy formation. Fe K-edge EXAFS reveals a Fe–O coordination number of 4.9 ± 0.4 with no detectable Fe–Fe scattering, confirming that Fe is atomically incorporated into the Zr–*oxo* nodes in mixed-valence Fe^2+^/Fe^3+^ forms, rather than existing as separate Fe oxide phases.

High-resolution O 1*s* spectra obtained via XPS (Fig. 2H) are separated into 2 peaks with binding energies of 531.5 and 533.1 eV, which are related to lattice oxygen (O_L_) and oxygen vacancy (O_V_), respectively [37]. Peak area analysis (Fig. S8) shows that the percentage of OV is markedly increased in OVs-Zr/Fe UiO-66-NH_2_ in comparison with the pristine sample because of the introduction of defects and lattice distortions caused by Fe^2+^/Fe^3+^ ions. In order to further confirm the existence of OVs, electron paramagnetic resonance (EPR) spectroscopy was performed. As shown in Fig. S9, a pronounced EPR signal at *g* ≈ 2.003 is observed for both materials, signature of unpaired electrons associated with OVs. While this binding-energy region may also contain contributions from hydroxyl groups or adsorbed species, the consistent correlation with other defect-sensitive techniques supports our interpretation. Notably, the signal is much stronger in OVs-Zr/Fe UiO-66-NH_2_ than in UiO-66-NH_2_, pointing to the formation of abundant oxygen vacancies after Fe incorporation. These OV sites should trap electrons to curb charge recombination, and also provide coordination sites for uranyl adsorption, so that the photocatalytic uranium reduction performance is improved synergistically.

### Optoelectronic properties

**Fig. 3.**
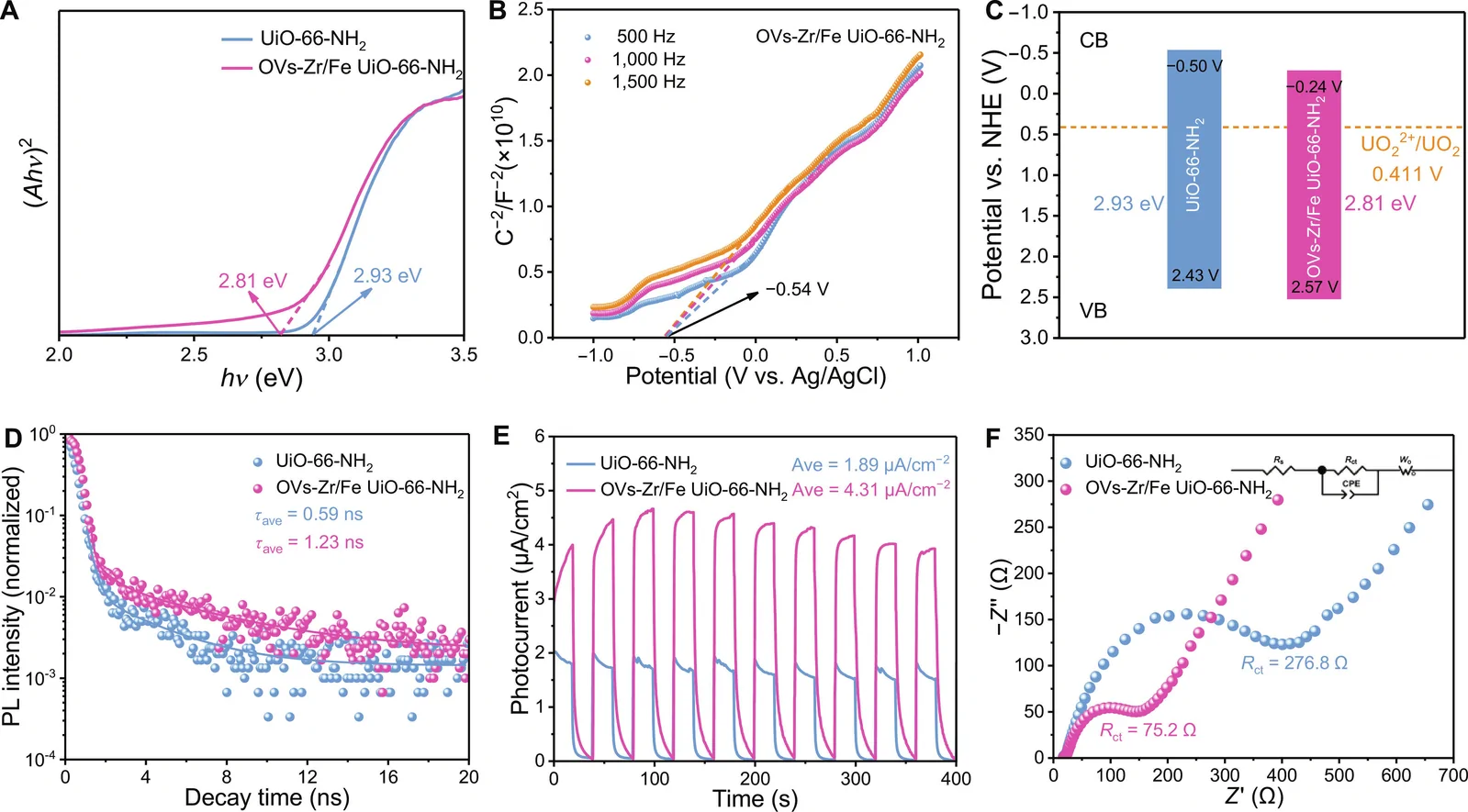
Optical and photoelectronic properties of UiO-66-NH_2_ and OVs-Zr/Fe UiO-66-NH_2_. (A) Tauc plot for bandgap calculation. (B) Mott–Schottky plots. (C) Band structure alignments. CB, conduction band; VB, valence band. NHE, normal hydrogen electrode. (D) Time-resolved photoluminescence (PL) decay spectra. (E) Transient current density. (F) Nyquist plots. The inset shows the equivalent circuit. CPE, constant phase element.

To compare their photocatalytic performance, we measured the optical absorption and electronic structure of pristine UiO-66-NH_2_ and OVs-Zr/Fe UiO-66-NH_2_. Diffuse reflectance spectroscopy revealed that the absorption cut-off wavelengths of UiO-66-NH_2_ and OVs-Zr/Fe UiO-66-NH_2_ are located at 447 and 485 nm, respectively, with the latter exhibiting a slightly extended absorption into the visible region (Fig. S10). The derived band gap (*E*_g_) narrowed from 2.93 eV for UiO-66-NH_2_ to 2.81 eV for OVs-Zr/Fe UiO-66-NH_2_ (Fig. 3A), indicating enhanced light-harvesting capability in the bimetallic system. Mott–Schottky analysis identified both as n-type semiconductors, with flat-band potentials (*E*_FB_) of −0.80 and −0.54 V (vs. Ag/AgCl and saturated KCl), respectively (Fig. S11 and Fig. 3B), which correspond to −0.60 and −0.34 V (vs. the normal hydrogen electrode, NHE). For n-type semiconductors, the conduction band edge is typically located approximately 0.1 V higher than the flat-band potential [38]. Notably, the conduction band (*E*_CB_) potential of OVs-Zr/Fe UiO-66-NH_2_ (−0.24 V vs. NHE) is more negative than the reduction potential of UO_2_^2+^/UO_2_ (0.411 V vs. NHE), providing the necessary thermodynamic force enabling the photocatalytic reduction of uranium (Fig. 3C). The corresponding valence band (*E*_VB_) potentials were calculated as 2.43 and 2.57 V (vs. NHE) for UiO-66-NH_2_ and OVs-Zr/Fe UiO-66-NH_2_, respectively. After correcting for the pH effect (pH 6.0), the conduction band of OVs-Zr/Fe UiO-66-NH_2_ is located at −0.595 V versus NHE, which is much more negative than the UO_2_^2+^/UO_2_ redox potential (0.056 V vs. NHE at pH 6.0), confirming the thermodynamic feasibility of the photoreduction process (Fig. S12). The incorporation of Fe into the UiO-66-NH_2_ framework modulates its electronic structure by introducing intermediate states via Fe 3*d*-ligand orbital hybridization and generating oxygen vacancies through lattice distortion. These effects collectively narrow the bandgap and extend visible-light absorption while maintaining conduction band potential sufficiently negative for U(VI) reduction.

The improved charge dynamics are attributed to the combination of a narrowed bandgap and oxygen vacancies that act as electron sinks. In this process, the Fe^2+^/Fe^3+^ couple serves as the primary mediator for charge transfer. Optoelectronic characterizations further support this mechanism. As shown in the steady-state photoluminescence (PL) spectra (Fig. S13), the PL intensity of OVs-Zr/Fe UiO-66-NH_2_ is quenched compared to the pristine MOF, suggesting the suppression of charge-carrier recombination. Time-resolved PL decay analysis revealed a prolonged average carrier lifetime (*τ*_avg_) from 0.59 to 1.23 ns (Fig. 3D), further confirming the suppression of charge recombination. This 2.1-fold prolongation points to the role of Fe^2+^/Fe^3+^ sites in capturing photogenerated electrons and shuttling them away from recombination centers.

The enhanced bulk charge separation facilitates interfacial charge transfer. Transient photocurrent measurements showed that the bimetallic OVs-Zr/Fe UiO-66-NH_2_ delivers an average photocurrent density of 4.31 μA/cm^2^, a 2.28-fold increase over the pristine material (1.89 μA/cm^2^), indicating improved charge extraction efficiency (Fig. 3E). In parallel, electrochemical impedance spectroscopy showed a decrease in charge-transfer resistance (*R*_ct_) from 276.8 to 75.2 Ω (Fig. 3F). The 3.7-fold reduction in *R*_ct_ suggests that the Fe^2+^/Fe^3+^ redox cycling enhances interfacial charge transfer. The combined effects of bandgap narrowing, oxygen-vacancy trapping, and Fe^2+^/Fe^3+^-mediated charge transfer collectively account for the enhanced photocatalytic performance [39]. These results demonstrate that the improved charge dynamics originate from the cooperative interplay between Fe substitution and the associated oxygen vacancies, rather than from either factor alone.

### Adsorption and photocatalytic performance

**Fig. 4.**
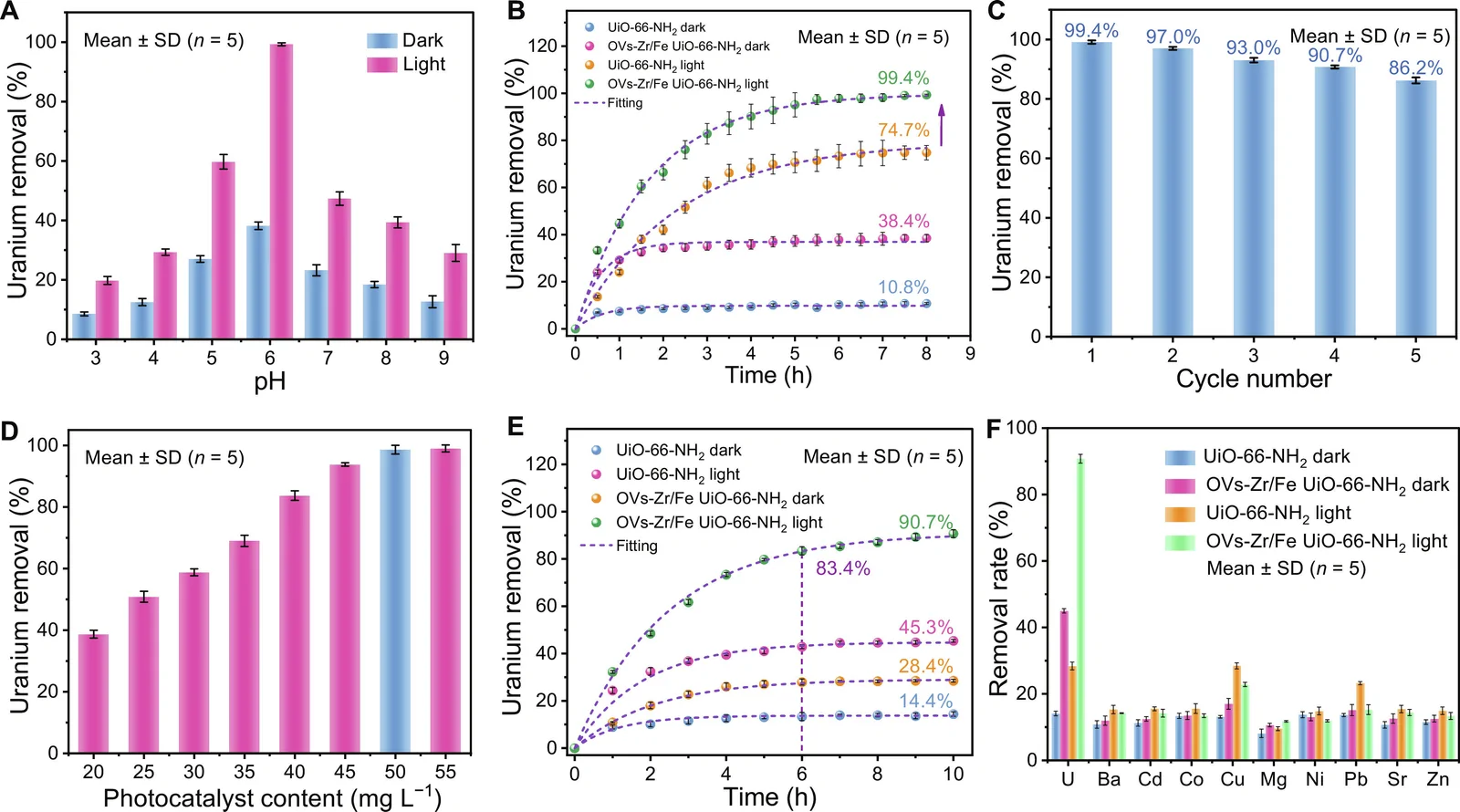
Photocatalytic performance of OVs-Zr/Fe UiO-66-NH_2_ toward uranium. (A) Influence of pH on photocatalytic ability under dark and light conditions. (B) U(VI) removal ratio in the dark and under one-sun irradiation. (C) Reusability of OVs-Zr/Fe UiO-66-NH_2_. (D) Influence of photocatalyst content, (E) kinetics of photocatalytic uranium removal, and (F) uranium removal selectivity of materials in the simulated multication-containing solution.

The adsorption and photocatalytic abilities of OVs-Zr/Fe UiO-66-NH_2_ toward uranium removal have been investigated at ambient conditions in the absence of any scavenging agent. In addition to the pH-dependent surface charge and uranium speciation, they dictate the immobilization ability of the catalyst. It can be seen in Fig. 4A that near-neutral pH values result in the best removal of uranium, where the maximum efficiency was attained at pH 6. Such behavior allows considering the material very promising for the practical application in the tailings wastewater purification [40]. U(VI) uptake was consistently higher under visible-light irradiation than in the dark at the same pH, suggesting that photocatalysis contributes additional removal beyond adsorption.

Under dark conditions, OVs-Zr/Fe UiO-66-NH_2_ exhibited a higher U(VI) adsorption capacity than UiO-66-NH_2_, though both materials showed limited removal efficiency. Removal efficiency increased from 38.4% in the dark to 99.4% under irradiation, 1.33 times higher than the 74.7% removal by pristine UiO-66-NH_2_ (Fig. 4B), suggesting that photoreduction is mainly responsible for the enhanced removal. Within 6 h of illumination, a maximum adsorption capacity of 375.90 mg g^−1^ was achieved (Fig. S14). Kinetic fitting with the pseudo-first-order model at 25 °C gave a rate constant (*k*) of 0.66 h^−1^ for OVs-Zr/Fe UiO-66-NH_2_, 1.78 times higher than the 0.37 h^−1^ for pristine UiO-66-NH_2_ (Fig. S15). The corresponding correlation coefficients (*R*^2^) were 0.994 and 0.991, respectively, indicating excellent agreement with the pseudo-first-order model (Table S3). The photocatalyst’s stability and reusability were evaluated by performing 5 sequential reaction cycles. As shown in Fig. 4C, OVs-Zr/Fe UiO-66-NH_2_ maintained 86.2% U(VI) removal efficiency in the fifth cycle using an 18.9-ppm uranium solution, corresponding to 86.7% retention of the initial capacity (99.4%), with good elution efficiency as well (Fig. S16). Post-cycling scanning electron microscopy, XRD, XPS, and EPR analyses (Fig. S17) confirmed that the morphology, crystalline framework, and the characteristic oxygen-vacancy-related EPR signal (*g* ≈ 2.003) were both well preserved after 5 cycles. These results indicate that the oxygen vacancies are stable under the reaction conditions, and the slight decrease in removal efficiency is primarily due to the gradual occupation of active sites by residual uranium species rather than structural degradation.

The uranium extraction capability of OVs-Zr/Fe UiO-66-NH_2_ was further evaluated using the simulated multication-containing solution (composition detailed in Table S4). As shown in Fig. 4D, the U(VI) removal efficiency increased with increasing adsorbent dosage. At pH 6, a dosage of 50 mg L^−1^ was enough to achieve almost full removal of U(VI) from an 18.9-ppm solution. The complex composition and high salinity of effluents make selective U(VI) capture particularly important for practical applications [41]. When tested at a dosage of 50 mg L^−1^ under high-ionic-strength conditions, the material exhibited rapid U(VI) extraction, reaching 83.4% within the first 6 h and attaining an equilibrium removal of 90.7% after 10 h (Fig. 4E). The good selectivity of OVs-Zr/Fe UiO-66-NH_2_ toward uranium removal is supported by its *K*_d_ (1.96 × 10^5^ mL g^−1^), which is about 11.9 times higher than that of unmodified UiO-66-NH_2_ (*K*_d_ = 1.65 × 10^4^ mL g^−1^) (Table S5). The selective U(VI) uptake of this sample over competing metal ions is evident from the distinctly higher removal efficiency of uranium than of other metal ions (Fig. 4F). The high *K*_d_ value and selectivity of U(VI) uptake over competing ions indicate the suitability of OVs-Zr/Fe UiO-66-NH_2_ for uranium extraction from complicated waste solutions. To further evaluate the feasibility of the material for practical wastewater treatment, we compared the residual uranium concentration after adsorption against the national regulatory standard for uranium mining and metallurgy facilities. According to the Chinese national standard (Regulations for radiation protection and radiation environment protection in uranium mining and milling), the discharge limit for natural uranium at the wastewater outlet is 0.3 mg L^−1^. In adsorption experiments using uranium tailings wastewater with an initial uranium concentration of approximately 0.5 mg L^−1^ (detailed parameters are shown in the Table S6) [42], the residual uranium concentration after treatment with OVs-Zr/Fe UiO-66-NH2 decreased to approximately 0.22 mg L^−1^, which is below the prescribed discharge limit of 0.3 mg L^−1^ (Fig. S18). Although the removal efficiency under complex low concentration matrix conditions is lower than that achieved in the simplified solution, the residual uranium concentration remains below the statutory discharge limit, indicating that the material retains good practical utility under complex working conditions.

### Mechanism analysis

**Fig. 5.**
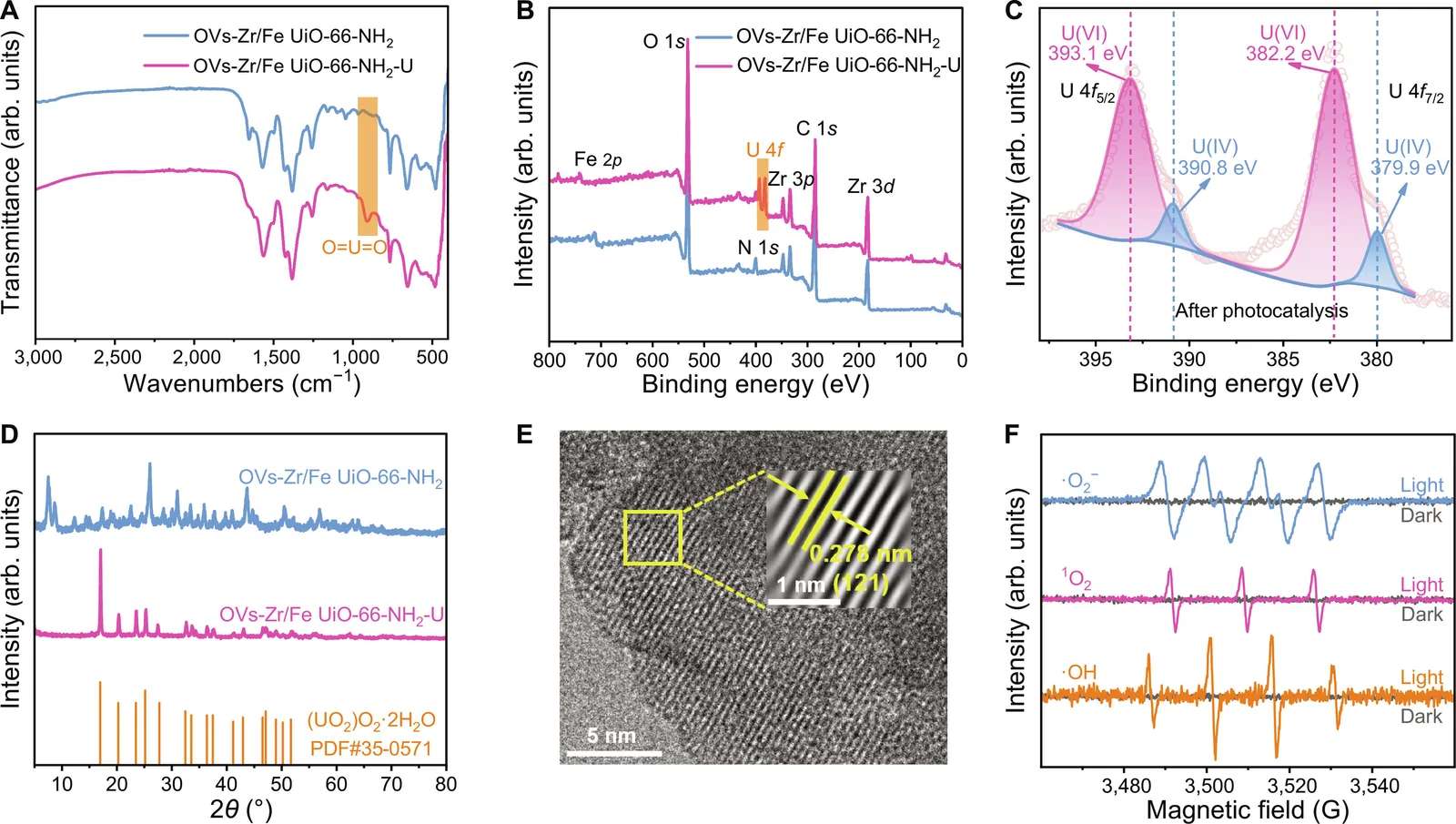
Photocatalytic mechanism of uranium by OVs-Zr/Fe UiO-66-NH_2_. (A) FT-IR spectra and (B) XPS spectra of OVs-Zr/Fe UiO-66-NH_2_ before and after uranium photocatalysis. (C) High-resolution XPS spectra of U 4*f* of the samples after photocatalysis. (D) XRD patterns of the OVs-Zr/Fe UiO-66-NH_2_, the photocatalytic products, and the standard Powder Diffraction File (PDF) card for (UO_2_)O_2_·2H_2_O. (E) High-resolution transmission electron microscopy image of the samples after photocatalysis. (F) Electron paramagnetic resonance spectra of the reactive oxygen species (·O_2_^−^, ^1^O_2_, and ·OH) in the solution before and after light exposure.

The photocatalytic immobilization mechanisms were studied by characterizing the recovered species. FT-IR spectrum of the post-reaction OVs-Zr/Fe UiO-66-NH_2_ showed characteristic bands of the framework while presenting a new vibration peak at 925 cm^−1^ attributed to *ν*(O=U=O) (Fig. 5A), proving uranium surface fixation [43]. Complementary energy dispersive spectroscopy (EDS) elemental mapping demonstrated homogeneous spatial co-distribution of U with Fe, Zr, C, N, and O (Fig. S19), further evidencing uranium incorporation. XPS spectra were acquired to probe the chemical valence states of the recovered uranium. The U 4*f* characteristic peak was distinctly detectable following the photocatalytic treatment of uranium in OVs-Zr/Fe UiO-66-NH_2_ (Fig. 5B). The binding energies differ from those of U(VI) in UO_2_(NO_3_)_2_·6H_2_O (382.2/393.1 eV) (Fig. S20) [44]. Extra peaks at 379.9 eV (4*f*_7/2_) and 390.8 eV (4*f*_5/2_) suggest the presence of U(IV) (Fig. 5C). Spectral deconvolution gave a U(IV):U(VI) ratio of 21.2%:78.8%, with U(VI) as the main photocatalytic product. In comparison, the convoluted U 4*f* spectrum of the pristine UiO-66-NH_2_ under identical reaction conditions presented exclusively U(VI) species (100%) with no detectable U(IV) signals (Fig. S21).

XRD patterns showed reflections at 16.97°, 20.26°, and 23.45° (Fig. 5D), which match (UO_2_)O_2_·2H_2_O (Powder Diffraction File #35-0571) [45]. HRTEM images revealed 0.278-nm lattice fringes, corresponding to the (121) plane of (UO_2_)O_2_·2H_2_O (Fig. 5E). These results indicate the formation of crystalline (UO_2_)O_2_·2H_2_O on the catalyst surface. To directly validate the involvement of the Fe^2+^/Fe^3+^ redox cycle, we performed time-resolved XPS measurements to track the evolution of Fe valence states. Initially, the Fe^2+^/Fe^3+^ ratio was 56.56%:43.44% (Fig. S22A). After 30 min of visible-light irradiation in pure water, the Fe^2+^/Fe^3+^ ratio increased distinctly to 66.77%:33.23% (Fig. S22B), confirming that photogenerated electrons reduce Fe^3+^ to Fe^2+^. Subsequently, upon introduction of UO_2_^2+^ and equilibration in the dark, the Fe^2+^ concentration sharply decreased, yielding a final Fe^2+^/Fe^3+^ ratio of 39.07%:60.93% (Fig. S22C). This dynamic evolution clearly demonstrates that Fe^3+^ captures photogenerated electrons to form Fe^2+^, which then donates electrons for U(VI) reduction and is re-oxidized back to Fe^3+^, thereby establishing a reversible redox cycle. Under these conditions, the generation of H_2_O_2_ was nearly not detected (Table S7).

EPR analysis detected ·O_2_^−^, ^1^O_2_, and ·OH during photocatalysis (Fig. 5F). The quenching experiments were implemented by introducing AgNO_3_ for e^−^, *p*-benzoquinone for ·O_2_^−^, furfuryl alcohol for ^1^O_2_, and tertiary butanol for ·OH [20,21]. The concentration of each scavenger was 0.4 mmol L^−1^. The added volumes were kept minimal to avoid significant interference with the reaction system. The results showed that scavenging e^−^, ·O_2_^−^, and ^1^O_2_ suppressed U(VI) removal, whereas ·OH scavenging had little effect (Fig. S23). The observed suppression pattern provides supporting evidence for the involvement of these species. The results are consistent with e^−^, ·O_2_^−^, and ^1^O_2_ being the dominant reactive species, with e^−^ likely responsible for U(VI) reduction and ·O_2_^−^/^1^O_2_ for the formation of (UO_2_)O_2_·2H_2_O.

$$\text{Photocatalyst}+h\nu \to e^{-}+h^{+}$$ (1)

$$O_{2}+e^{-}\to \cdot {O_{2}}^{-}$$ (2)

$${\mathrm{Fe}}^{3+}+e^{-}\to {\mathrm{Fe}}^{2+}$$ (3)

$${\mathrm{Fe}}^{2+}+{{\mathrm{UO}}_{2}}^{2+}\to {\mathrm{Fe}}^{3+}+{{\mathrm{UO}}_{2}}^{+}$$ (4)

$${\mathrm{Fe}}^{2+}+{{\mathrm{UO}}_{2}}^{+}\to {\mathrm{Fe}}^{3+}+{\mathrm{UO}}_{2}$$ (5)

$${{\mathrm{UO}}_{2}}^{+}+\cdot {O_{2}}^{-}+{\text{2H}}_{2}O\to \left({\mathrm{UO}}_{2}\right)O_{2}\cdot {\text{2H}}_{2}O$$ (6)

$${\mathrm{UO}}_{2}+O_{2}+{\text{2H}}_{2}O\to \left({\mathrm{UO}}_{2}\right)O_{2}\cdot {\text{2H}}_{2}O$$ (7)

$${\text{2H}}_{2}O+4h^{+}\to O_{2}+{\text{4H}}^{+}$$ (8)

The photocatalytic reduction is accomplished via the cooperation of 2 active sites. The first step involves doping of Fe to decrease the band gap of the materials, thus generating more photoelectrons upon irradiation with visible light. The photoelectrons will be accumulated in OV sites, which form reduced zones for capturing UO_2_^2+^. At the same time, the neighboring Fe^2+^/Fe^3+^ redox pair works as an efficient electron carrier, in which Fe^3+^ efficiently traps photogenerated electrons to give extremely reductive Fe^2+^, which further reduces the adjacent adsorbed UO_2_^2+^ to U(IV), recycling Fe^3+^ to complete the photocatalytic process. In parallel, the photogenerated holes migrate to the catalyst surface and are consumed through the oxidation of adsorbed H_2_O and OH^−^, producing ·OH and O_2_ that prevent charge accumulation. Meanwhile, dissolved O_2_ in the solution acts as an electron acceptor and is reduced to ·O_2_^−^, which further balances the charge and sustains the redox cycle. This coupled electron hole transfer pathway enables efficient and selective U(VI) removal under ambient air without the need for sacrificial agents. Although the initial photocatalytic step involves the reduction of U(VI) to U(IV), the final crystalline product identified by XRD and HRTEM is (UO_2_)O_2_·2H_2_O. This is because the photogenerated reactive oxygen species subsequently re-oxidize the reduced uranium intermediates, which then interact with ·O_2_^−^/O_2_ and H_2_O, eventually forming this stable hydrated uranium peroxide phase under our aerobic, sacrificial-agent-free conditions. The 21.2% U(IV) species detected by XPS correspond to these partially reduced intermediates trapped on the catalyst surface before complete oxidation. This synergy mechanism may rely on the cooperative roles of OVs sites for concentrating reactants and electrons and the Fe^2+^/Fe^3+^ cycling controlling the crucial interfacial electron transfer.

**Fig. 6.**
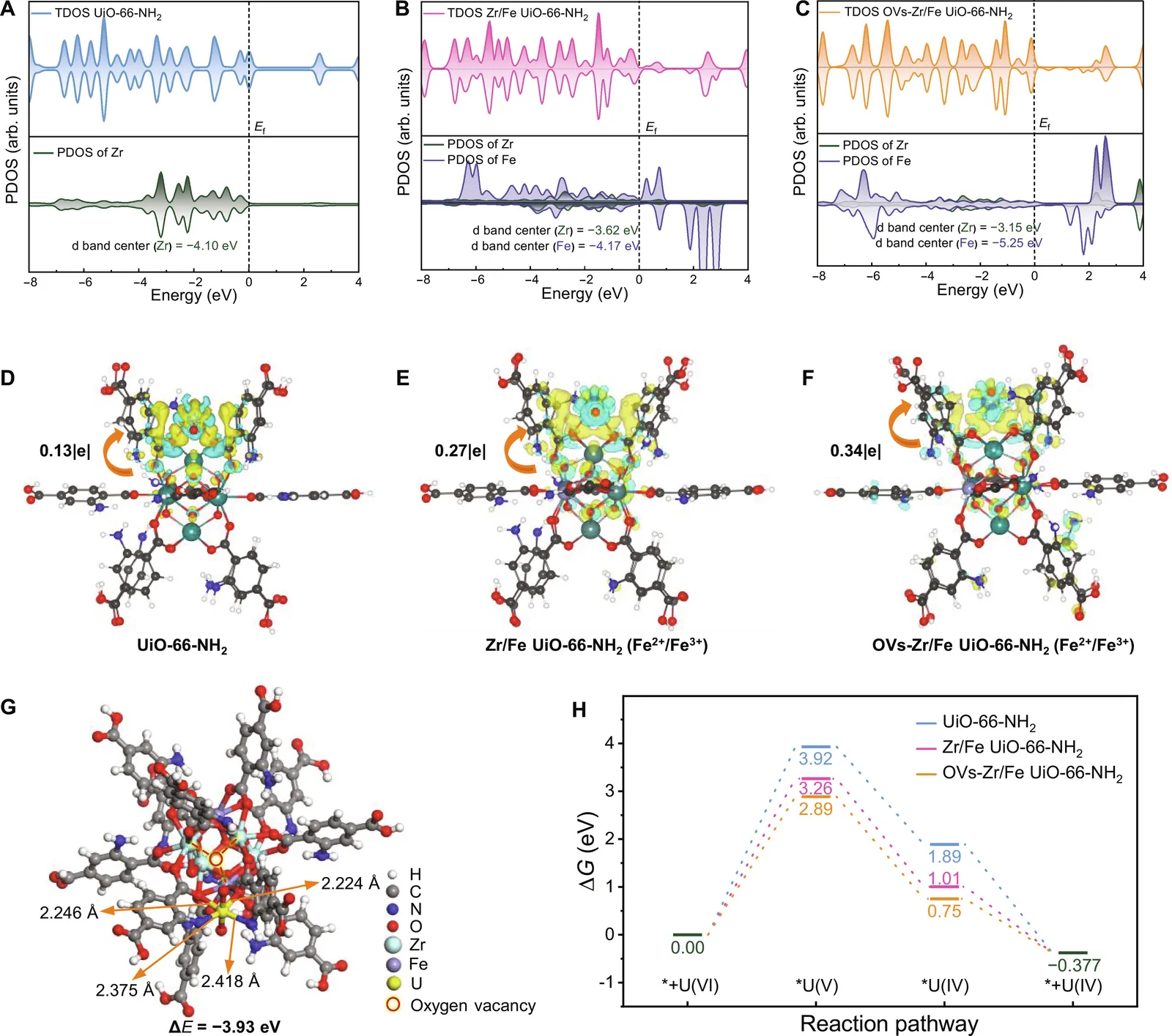
Density of states of the (A) UiO-66-NH_2_, (B) Zr/Fe UiO-66-NH_2_, and (C) OVs-Zr/Fe UiO-66-NH_2_. PDOS, partial density of states; TDOS, total density of states. Charge density difference iso-surfaces of the (D) UiO-66-NH_2_, (E) Zr/Fe UiO-66-NH_2_, and (F) OVs-Zr/Fe UiO-66-NH_2_ at an iso-surface value of 0.003|e| Å^−3^. Yellow and cyan denote charge accumulation and depletion, respectively. (G) The optimized structures of OVs-Zr/Fe UiO-66-NH_2_ and DFT calculation of the binding energy. (H) Relative Gibbs-free-energy diagram for the reduction of uranium.

The adsorption of UO_2_^2+^ at catalytic sites is required for subsequent photoreduction. In order to investigate the atomistic mechanism involved, density functional theory (DFT) calculation was performed, in which the relation between electronic structure and uranyl adsorption and reduction was correlated. As illustrated by partial density of states/total density of states analysis, Fe addition results in new electronic states close to Fermi energy levels and d-band center movement of Zr from −4.10 eV in pristine UiO-66-NH_2_ to −3.62 eV in Zr/Fe UiO-66-NH_2_ (Fig. 6A and B). After OV generation, the electronic structure is further regulated, the d-band center being moved to −3.15 eV with localized defect states emerging in OVs-Zr/Fe UiO-66-NH_2_ (Fig. 6C). These observations suggested that accelerated electron transfer and easier access of electrons to active sites may be due to Fe/OV regulation. Through charge density difference calculation, the charge distribution in active regions is further investigated (Fig. 6D to F). As can be seen, the built-up charges rise from 0.13|e| for UiO-66-NH_2_ to 0.27|e| for Zr/Fe UiO-66-NH_2_ and 0.34|e| for OVs-Zr/Fe UiO-66-NH_2_, indicating an enhancement of the local electronic field due to Fe loading and OV formation. Electrostatic potential calculations further corroborate this trend. Upon introduction of oxygen vacancies, the charge distribution becomes increasingly uneven, with a substantially expanded negative electrostatic potential region, which is favorable for the electrostatic adsorption of positively charged UO_2_^2+^ (Fig. S24) [46]. The optimized adsorption configuration and binding-energy calculations show stronger uranyl affinity on the modified framework. The adsorption energy of UO_2_^2+^ on OVs-Zr/Fe UiO-66-NH_2_ is −3.93 eV, more negative than that on pristine UiO-66-NH_2_ (−3.16 eV), indicating the enhanced interaction between UO_2_^2+^ and the OV-regulated local coordination environment (Fig. 6G and Fig. S25).

The OV–Fe coupled structure also promotes the reduction process. The relative Gibbs-free-energy diagram shows that the free-energy barrier for forming the key U(V) intermediate on OVs-Zr/Fe UiO-66-NH_2_ is reduced to 2.89 eV, much lower than that on pristine UiO-66-NH_2_ (3.92 eV; Fig. 6H). This decrease can be attributed to the cooperative roles of OVs and Fe^2+^/Fe^3+^ sites, where OVs enrich electrons at the adsorption sites and Fe^2+^/Fe^3+^ relay these electrons to the coordinated UO_2_^2+^, lowering the energy barrier for U(VI) reduction.

## Conclusion

In summary, we designed a bimetallic OVs-Zr/Fe UiO-66-NH_2_ photocatalyst for selective U(VI) photoreduction and immobilization in complex aqueous systems. Fe doping narrowed the bandgap, improved visible-light utilization, and promoted the formation of Fe^2+^/Fe^3+^ redox couples and oxygen vacancies. Together with the amino groups and hierarchical pore structure, the oxygen vacancies provide uranyl adsorption sites and electron traps, which collectively contribute to selective UO_2_^2+^ enrichment and charge separation. At the same time, the redox couple between the neighboring Fe^2+^/Fe^3+^ allows electrons to be transferred to the uranyl ions in OV-modified sites, thereby achieving U(VI) reduction. By means of the synergistic effect of these 2 mechanisms, OVs-Zr/Fe UiO-66-NH_2_ was able to remove 99.2% of U(VI) in 6 h under ambient air without any sacrificial reagents, and its *K*_d_ increased to 1.96 × 10^5^ ml g^−1^, 11.9 times that of pristine UiO-66-NH_2_. The high *K*_d_ value and its consistent U(VI) removal under the conditions of competitive cations indicate selectivity of the material toward uranium in complicated wastewater. The synergy between oxygen vacancies and Fe^2+^/Fe^3+^ redox cycles offers a pathway for designing MOF-based photocatalysts for uranium remediation.

## Materials and Methods

### Materials

*N*,*N*-Dimethylformamide (DMF, 99.9%) and acetic acid (CH_3_COOH, 99.7%) were obtained from Xilong Scientific Co., Ltd. Iron chloride hexahydrate (FeCl_3_·6H_2_O, 99.0%) was purchased from Ron Chemical Co., Ltd. Ascorbic acid was purchased from Shanghai Yuanye Biotechnology Co., Ltd. Zirconium (IV) chloride (ZrCl_4_, 98.0%), 2-aminoterephthalic acid (H_2_BDC-NH_2_, 98.0%), uranium nitrate hexahydrate (UO_2_(NO_3_)_2_·6H_2_O, 99%), and Arsenazo III (95%) were obtained from Shanghai Macklin Biochemical Technology Co., Ltd. All commercial reagents were used without any further treatment.

### Synthesis of OVs-Zr/Fe UiO-66-NH_2_

The bimetallic MOF OVs-Zr/Fe UiO-66-NH_2_ was prepared using a procedure analogous to that used for UiO-66-NH_2_. ZrCl_4_ (0.0932 g, 0.4 mmol), FeCl_3_·6H_2_O (0.0541 g, 0.2 mmol), and acetic acid (4.0 ml) were successively added to 16.0 ml of anhydrous DMF under stirring for 20 min. Subsequently, ascorbic acid (0.0176 g, 0.1 mmol) was introduced into the mixture and stirred for 20 min to reduce Fe^3+^ to Fe^2+^. Then, H_2_BDC-NH_2_ (2-aminoterephthalic acid, 0.1087 g, 0.6 mmol) was dissolved in the resulting mixture under sonication for 20 min. The solution was poured into a 100-ml autoclave and then heated at 120 °C for a duration of 24 h. After cooling naturally to room temperature, the product was collected by centrifugation and repeatedly washed 3 times with DMF and anhydrous ethanol to remove any residual reactants. Finally, the obtained OVs-Zr/Fe UiO-66-NH_2_ powder got dried under vacuum at 60 °C for 24 h.

### Uranium removal ability assay

Photocatalytic tests were carried out under simulated solar irradiation (300-W Xe lamp, 200 to 800 nm, 1 kW·m^−2^). A 100-ml aliquot of uranium solution (18.9 ppm, pH 6) containing 5 mg of catalyst was taken at fixed time intervals, passed through a 0.22-μm filter, and analyzed by the Arsenazo III spectrophotometric method. Selectivity experiments were performed in s simulated multication-containing solution (pH 6.0, Table S5) with the same catalyst loading. Metal ion concentrations in the supernatant were determined by inductively coupled plasma optical emission spectrometry, and the uranium removal rate (*C*) was calculated using Eq. (9):

$$C=\frac{\left(C_{0}-C_{e}\right)}{C_{0}}\times 100\%$$ (9)

where *C*_0_ is the initial uranium concentration in solution (mg L^−1^) and *C*_e_ is the equilibrium uranium concentration (mg L^−1^).

The distribution coefficient *K*_d_ (mL g^−1^) is defined as the ratio of the amount of uranium adsorbed on the solid phase (per gram of adsorbent) to the concentration of uranium remaining in the liquid phase at equilibrium, according to the following Eq. (10):

$$K_{d}=\frac{\left(C_{0}-C_{e}\right)}{C_{e}}\times \frac{V}{m}$$ (10)

where *V* is the volume of the solution (ml) and *m* is the mass of the adsorbent (g). This equation yields *K*_d_ in units of milliliters per gram, which is the conventional unit used for comparing sorption selectivity.

Reusability tests were performed with 5 mg of photocatalyst in 100 ml of uranium solution (18.9 ppm, pH 6.0). Following 12 h of Xe-lamp irradiation, the catalyst was collected by centrifugation and subsequently soaked in 0.1 mol/L HNO_3_ with 1 h of ultrasonication to strip the adsorbed uranium. The elution efficiency (EE) was derived from Eq. (11):

$$\mathrm{EE}=\frac{\left(C_{E}\times V_{E}\right)}{W_{E}}\times 100\%$$ (11)

where *C*_E_ is the eluted uranium concentration (ppm), *W*_E_ is the adsorbed U mass (mg), and *V*_E_ is the eluent volume (L).

## Data Availability

All relevant data are provided in the main manuscript and its supplementary material. The corresponding author will supply any additional datasets upon request.
