# Supplementary material for: Oxygen Vacancy–Redox Synergy in a Zr/Fe Metal–Organic Framework Enables Highly Selective Uranyl Photoreduction and Immobilization
Source: Research (Wash D C). 2026 Sep 9;9:1425. doi: 10.34133/research.1425 (PMC13554414; doi:10.34133/research.1425)
Supplement: Supplementary file 1 [file research.1425.f1.docx]

Supplementary Materials for

**Oxygen Vacancy-Redox Synergy in a Zr/Fe Metal-Organic Framework Enables Highly Selective Uranyl Photoreduction and Immobilization**

Taohong Xu ^1, 2, #^, Lijuan Feng ^1, 2, #^, Peng Liu ^1, 2^, Xinhang Zhao ^1, 2^, Hui Wang ^1, 2, *^, Guanbing Zhou ^1, 2, *^, Ning Wang ^1, 2, *^

^1^ School of Marine Sciences, State Key Laboratory of Marine Resource Utilization in South China Sea, Hainan University, Haikou 570228, China

^2^ State Key Laboratory of Tropic Ocean Engineering Materials and Materials Evaluation, Hainan University, Haikou 570228, China

^*^ Corresponding author.

*E-mail address*: wangn02@foxmail.com (N. Wang), [zhouguanbing@hainanu.edu.cn](mailto:zhouguanbing@hainanu.edu.cn) (G. Zhou), [huiw0318@hainanu.edu.cn](mailto:huiw0318@hainanu.edu.cn) (H. Wang)

^#^ These authors contributed equally to this work.

Contents

Preparation of UiO-66-NH_2_	4

Characterization methods	5

Supplementary DFT method	7

Supplementary Figures	8

Figure S1. The HR-TEM images of UiO-66-NH_2_ and OVs-Zr/Fe UiO-66-NH_2_	8

Figure S2. The EDX mapping of OVs-Zr/Fe UiO-66-NH_2_	9

Figure S3. XANES and FT‑EXAFS spectra.	10

Figure S4. Zr K-edge EXAFS fitting results for pristine UiO-66-NH_2_	11

Figure S5. Zr K-edge EXAFS fitting results for OVs-Zr/Fe UiO-66-NH_2_	12

Figure S6. Normalized Fe K-edge XANES spectra of OVs-Zr/Fe UiO-66-NH_2_, along with Fe foil, Fe_2_O_3_, and FeO references	13

Figure S7. Fe K-edge EXAFS analysis of OVs-Zr/Fe UiO-66-NH_2_	14

Figure S8. The relative percentage of different oxygen species	15

Figure S9. EPR spectra	16

Figure S10. The diffuse reflectance spectroscopy (DRS) of UiO-66-NH_2_ and OVs-Zr/Fe UiO-66-NH_2_	17

Figure S11. Mott-Schottky plots of UiO-66-NH_2_	18

Figure S12. Energy band diagram of UiO‑66‑NH_2_ and OVs‑Zr/Fe UiO‑66‑NH_2_ at pH 6.0 (vs. NHE).	19

Figure S13. Steady-state photoluminescence (PL) spectra of UiO-66-NH_2_ and OVs-Zr/Fe UiO-66-NH_2_	20

Figure S14. U(VI) uptake capacity of UiO-66-NH_2_ and OVs-Zr/Fe UiO-66-NH_2_ in the dark and under one-sun irradiation	21

Figure S15. The pseudo-first-order model at 25°C of UiO-66-NH2 and OVs-Zr/Fe UiO-66-NH_2_ in the dark and under one-sun irradiation	22

Figure S16. The elution rate of OVs-Zr/Fe UiO-66-NH_2_ within five cycles	23

Figure S17. The characterization of OVs-Zr/Fe UiO-66-NH_2_ after five photocatalytic cycles	24

Figure S18. Variation of uranium concentration in real uranium tailings wastewater before and after treatment at solid-liquid ratio of 0.05 g L^–1^ and pH = 6	25

Figure S19. EDS elemental mapping of OVs-Zr/Fe UiO-66-NH_2_ after uranium photocatalysis	26

Figure S20. High-resolution XPS spectra of U 4*f* for UO_2_(NO_3_)_2_·6H_2_O	27

Figure S21. High-resolution XPS spectra of U 4*f* for UiO-66-NH_2_ after uranium removal under light conditions	28

Figure S22. Time-resolved high-resolution Fe 2*p* XPS spectra of OVs-Zr/Fe UiO-66-NH_2_ at different stages	29

Figure S23. Influences of various sacrificial agents on photocatalysis of uranium over OVs-Zr/Fe UiO-66-NH_2_	30

Figure S24. The ESP surface of the photocatalysis.	31

Figure S25. Density functional theory (DFT) calculations illustrating the adsorption and two-step reduction pathway of UO_2_^2+^ on photocatalysis, along with the corresponding binding energies.	32

Supplementary Tables	33

Table S1. Summary of porosity properties of UiO-66-NH_2_ and OVs-Zr/Fe UiO-66-NH_2_	33

Table S2. Structural parameters extracted from the EXAFS data fitting	34

Table S3. Fitting parameters of pseudo-second-order and pseudo-first-order models	36

Table S4. Formulation of the simulated multication-containing solution	37

Table S5. Details of the calculation of the *K*_d_ value	38

Table S6. Uranium tailings wastewater. Ion content analysis	39

Table S7. The generation of H_2_O_2_ under the light	40

**Preparation of UiO-66-NH_2_**

According to previously reported procedures with some modifications, UiO-66-NH_2_ crystals were synthesized as follows. ZrCl4 (0.1398 g, 0.6 mmol) and acetic acid (4.0 mL) were successively added into 16.0 mL of anhydrous DMF under stirring for 20 min. Subsequently, H2BDC-NH_2_ (2-aminoterephthalic acid, ATA, 0.1087 g, 0.6 mmol) was dissolved in the above mixture under sonication for 20 min. The resulting solution was then transferred into a 100 mL autoclave and heated at 120 °C for 24 h. The reaction mixture was left to cool to ambient temperature. The product was then collected by centrifugation and subjected to three washing cycles using DMF and anhydrous ethanol, ensuring complete removal of residual reactants. Finally, the obtained UiO-66-NH_2_ powder was vacuum-dried at 60 °C for 24 h.

**Characterization methods**

FT-IR transmission spectra were acquired using a Nicolet lS10 spectrometer with KBr pellets. The XRD pattern were obtained by the D8 ADVANCE X-ray diffractometer of Bruker, Germany. Surface morphology was examined using a Hitachi SU8020 field-emission scanning electron microscope (FESEM). The TEM, HR-TEM, EDS elemental mapping and lattice fringes were obtained from a FEI Talos F200X instrument. UV-3600 (Shimadzu) was used to measure absorption spectra and UV-Vis DRS. N_2_ physisorption isotherms were collected using a Micromeritics ASAP 2460 analyzer. Specific surface area was estimated by the BET method, and pore size distribution was analyzed via the NLDFT model. A Thermo ICP-OES 7200 (USA) was employed to quantify the concentrations of competing ions. XPS was conducted on a Thermo Escalab 250Xi instrument Al Kα (hν = 1486.6 eV) to probe surface chemistry and oxidation states. PL spectra (steady-state and time-resolved) were measured with an Edinburgh FLS1000 spectrometer. For photoelectrochemical characterization, we used a Chenhua CHI-760E workstation to record transient photocurrent, electrochemical impedance spectra, and Mott-Schottky curves. Electron paramagnetic resonance (EPR) measurements in X-band (microwave frequency ≈ 9.83 GHz) were performed at 293 K by a Bruker E500-10/12 micro spectrometer.

Photoelectric performance test

Photocurrent responses were recorded in 0.5 M Na_2_SO_4_ with a three-electrode system (FTO/photocatalyst as working, Pt as counter, Ag/AgCl as reference) on a CHI 760E workstation. Catalyst was prepared by dispersing the powder in ethanol containing 0.05 wt% Nafion, then spin-coated onto FTO glass (1.0 × 1.0 cm^2^). Mott-Schottky measurements were carried out at 500, 1000, and 1500 Hz. The photocurrent responses were conducted with a CHI 760E workstation, with the working electrodes irradiated from the front side. Steady-state photoluminescence (PL) emission spectra and PL decay spectra were measured at room temperature using FLS 1000 spectrometer (Edinburgh Instruments, UK). The light was generated by a 300 W xenon lamp (PLS-SXE300D) with a light density of 1 kW m^−2^ at room temperature with the light wavelength from 300 nm to 2500 nm. Electrochemical impedance spectroscopy (EIS) spectra were collected from 0.1 Hz to 100 kHz under dark conditions with an applied bias of 5 mV. All measured potentials were converted to the normal hydrogen electrode (NHE) scale using the following equation (S1):

|  | $\text{E}\text{ }\text{(vs. NHE)}\text{ }\text{=}\text{ }\text{E}\text{ (vs. Ag/AgCl, sat. KCl) + 0.197 V}\text{ }\text{(25℃)}$ | S1 |
| --- | --- | --- |

The test conditions for EPR

The test conditions for EPR. EPR measurements in X-band (microwave frequency ≈ 9.83 GHz) were performed at 293 K by a Bruker E500-10/12 micro spectrometer. The samples were illuminated by a 300 W Xe lamp with 420 nm cut-off filter (LOT Oriel). All the samples were measured under the same conditions (microeave power: 0.63 mW, modedulation frequency: 65 KHz, modulation amplitude: 1 G, sweep time 40 s). g values have been caculated from the resonance fied B_0_ and the resonance frequency ν using the resonance condition *hν* = gβB_0_.

**Supplementary DFT method**

First-principles calculations based on density functional theory (DFT) were performed utilizing the Vienna ab initio simulation package (VASP 6.2.1). The exchange-correlation energy was treated within the generalized gradient approximation (GGA) as parameterized by the Perdew-Burke-Ernzerhof (PBE) function. A cutoff energy of 500 eV was employed for the plane-wave basis set following rigorous convergence calculations. All the atoms in the models were fully relaxed during all the optimization. Ionic relaxation was performed until the energy difference between consecutive steps was below 0.01 meV and the Hellmann-Feynman forces acting on each atom were less than 0.01 eV·Å^−1^. The Brillouin zone was integrated using a Monkhorst-Pack scheme with a 1 × 1 × 1 k-point mesh for the separation of 0.05 Å^−1^. Periodic boundary conditions in the z-direction were mitigated by the inclusion of a 15 Å vacuum layer to decouple adjacent slabs. DFT+U method was utilized for Zr, Fe and U atoms with the U values of 4.0, 3.5 and 5.0, respectively. The models of MOF were cutting a node with 6 six metal atoms and 12 ligands (model1 contains 6 Zr atoms, model2 contains 4 Zr atoms and 2 Fe atoms). Model3 was built basing on model2 by removing 1 O atoms in Zr-Zr-Fe site (the only O environment beside ligand O). Solvation effects were incorporated via the VASPsol implicit solvation model (ε = 80).

**Supplementary Figures**

**Figure S1.** The HR-TEM images of UiO-66-NH_2_ and OVs-Zr/Fe UiO-66-NH_2_.

**Figure S2.** The EDX mapping of OVs-Zr/Fe UiO-66-NH_2_.

**Figure S3.** XANES and FT‑EXAFS spectra. (a) Normalized Zr K-edge XANES spectra and (b) *k*^2^-weighted Zr K-edge EXAFS Fourier transform (FT) magnitudes for pristine UiO-66-NH_2_ and OVs-Zr/Fe UiO-66-NH_2_.

**Figure S4.** Zr K-edge EXAFS fitting results for pristine UiO-66-NH_2_. (a) *k*^2^-weighted EXAFS FT spectra in R space (circles: experimental data, line: fitting curve). (b) Corresponding EXAFS oscillations in k space.

**Figure S5**. Zr K-edge EXAFS fitting results for OVs-Zr/Fe UiO-66-NH_2_. (a) *k*^2^-weighted EXAFS FT spectra in R space. (b) Corresponding EXAFS oscillations in k space.

**Figure S6**. Normalized Fe K-edge XANES spectra of OVs-Zr/Fe UiO-66-NH_2_, along with Fe foil, Fe_2_O_3_, and FeO references.

**Figure S7**. Fe K-edge EXAFS analysis of OVs-Zr/Fe UiO-66-NH_2_. (a) *k*^2^-weighted EXAFS FT spectra in R space for OVs-Zr/Fe UiO-66-NH_2_ and references. (b) EXAFS fitting results (R space, k space) for OVs-Zr/Fe UiO-66-NH_2_.

**Figure S8.** The relative percentage of different oxygen species.

**Figure S9.** EPR spectra.

**Figure S10.** The diffuse reflectance spectroscopy (DRS) of UiO-66-NH_2_ and OVs-Zr/Fe UiO-66-NH_2_.

**Figure S11.** Mott-Schottky plots of UiO-66-NH_2_.

**Figure S12.** Energy band diagram of UiO‑66‑NH_2_ and OVs‑Zr/Fe UiO‑66‑NH_2_ at pH 6.0 (vs. NHE).

**Figure S13.** Steady-state photoluminescence (PL) spectra of UiO-66-NH_2_ and OVs-Zr/Fe UiO-66-NH_2_.

**Figure S14.** U(VI) uptake capacity of UiO-66-NH_2_ and OVs-Zr/Fe UiO-66-NH_2_ in the dark and under one-sun irradiation.

**Figure S15.** The pseudo-first-order model at 25°C of UiO-66-NH_2_ and OVs-Zr/Fe UiO-66-NH_2_ in the dark and under one-sun irradiation.

**Figure S16.** The elution rate of OVs-Zr/Fe UiO-66-NH_2_ within five cycles.

**Figure S17.** The characterization of OVs-Zr/Fe UiO-66-NH_2_ after five photocatalytic cycles. (a) SEM image. (b) XRD. (c) XPS spectra. (d) EPR spectra.

**Fig. S18.** Variation of uranium concentration in real uranium tailings wastewater before and after treatment at solid-liquid ratio of 0.05 g L^–1^ and pH = 6 (Mean ± SD, n=3; the red dashed line represents the discharge standard limit).

**Figure S19.** EDS elemental mapping of OVs-Zr/Fe UiO-66-NH_2_ after uranium photocatalysis.

**Figure S20.** High-resolution XPS spectra of U 4*f* for UO_2_(NO_3_)_2_·6H_2_O.

**Figure S21.** High-resolution XPS spectra of U 4*f* for UiO-66-NH_2_ after uranium removal under light conditions.

**Figure S22.** Time-resolved high-resolution Fe 2*p* XPS spectra of OVs-Zr/Fe UiO-66-NH2 at different stages: (a) initial state (0 h); (b) after 30 min of visible-light irradiation in pure water; (c) after subsequent introduction of UO₂²⁺ and equilibration in the dark.

**Figure S23.** Influences of various sacrificial agents on photocatalysis of uranium over OVs-Zr/Fe UiO-66-NH_2_.

**Figure S24.** The ESP surface of the (a) UiO-66-NH_2_, (b) Zr/Fe UiO-66-NH_2_ and (c) OVs-Zr/Fe UiO-66-NH_2_.

**Figure S25.** Density functional theory (DFT) calculations illustrating the adsorption and two-step reduction pathway of UO_2_^2+^ on (a) UiO-66-NH_2_, (b) Zr/Fe UiO-66-NH_2_, and (c) OVs-Zr/Fe UiO-66-NH_2_, along with the corresponding binding energies.

**Supplementary Tables**

**Table S1. Summary of porosity properties of UiO-66-NH_2_ and OVs-Zr/Fe UiO-66-NH_2_.**

| **Sample** | **S_BET_ (m^2^/g)** | **V_micro_ (cm^3^/g)** | **V_meso_ (cm^3^/g)** | **V_total_ (cm^3^/g)** |
| --- | --- | --- | --- | --- |
| UiO-66-NH_2_ | 1061.3 | 0.3899 | 0.0446 | 0.4345 |
| OVs-Zr/Fe UiO-66-NH_2_ | 830.4 | 0.2298 | 0.2879 | 0.5177 |

**Table S2. Structural parameters extracted from the EXAFS data fitting.**

| Sample | Shell | *CN^[a]^* | R (Å)^[b]^ | σ^2^×10^-3^ (Å^2^) *^[c]^* | R-range/Å | *ΔE^[d]^* (eV) | *R^[e]^* factor |
| --- | --- | --- | --- | --- | --- | --- | --- |
| Zr foil | Zr-Zr | 6* | 3.14±0.01 | 1.5±0.6 | 1.8-3.6 | -3.4±0.8 | 0.007 |
|  | Zr-Zr | 6* | 3.26±0..01 | 1.6±0.2 |  |  |  |
| UiO-66-NH_2_-Zr | Zr-O | 8.3±0.8 | 2.23±0.01 | 6.0±0.9 | 1-4 | 2.6±1.5 | 0.019 |
| OVs-Zr/Fe UiO-66-NH_2_-Zr | Zr-O | 7.7±0.7 | 2.23±0.01 | 5.7±0.9 | 1-4 | 3.1±1.2 | 0.014 |
| Fe foil | Fe-Fe | 8* | 2.46±0.01 | 5.1±1.8 | 1-3 | 3.7±1.8 | 0.008 |
| OVs-Zr/Fe UiO-66-NH_2_-Fe | Fe-O | 4.9±0.4 | 2.0±0.01 | 9.1±1.4 | 1-2.1 | -1.4±0.8 | 0.005 |
| Fe_2_O_3_ | Fe-O | 5.4±0.2 | 2.01±0.01 | 2.9±0.3 | 1-3 | -3.2±0.6 | 0.003 |
|  | Fe-O-Fe | 11.3±0.1 | 2.95±0.03 | 5.2±1.9 |  |  |  |
| FeO | Fe-O | 5.4±0.5 | 2.09±0.02 | 5.7±2.6 | 1-3 | -4.1±1.7 | 0.013 |
|  | Fe-O-Fe | 10.2±0.4 | 3.05±0.02 | 6.1±1.6 |  |  |  |

[a] *CN*: Mnordination numbers; [b] *R*: bond distance; [c] *σ*^2^: Debye-Waller factors; [d] Δ*E*_0_: the inner potential Mnrrection. [e] R factor: goodness of fit. Error bounds that characterize the structural parameters obtained by EXAFS spectrosMnpy were estimated as *CN*±20%; *R* ± 1%; *σ^2^* ± 20%. EXAFS fitting parameters at Zr K-edge (S_0_^2^ = 0.78). EXAFS fitting parameters at Fe K-edge (S_0_^2^ = 0.71)

The obtained XAFS data was processed in Athena (version 0.9.25) for background, pre-edge line and post-edge line calibrations. Then Fourier transformed fitting was carried out in Artemis (version 0.950). The k^2^ weighting, k-range of 3 – 11 Å^-1^ and R range of 1~3 Å were used for the fitting. The four parameters, coordination number, bond length, Debye-Waller factor and E0 shift (CN, R, σ^2^, ΔE_0_) were fitted without anyone was fixed, constrained, or correlated.

For Wavelet Transform analysis, the χ(k) exported from Athena was imported into the Hama Fortran code. The parameters were listed as follow: R range, 0 - 6 Å, k range, 0 - 15 Å^-1^; k weight, 2; and Morlet function with κ=10, σ=1 was used as the mother wavelet to provide the overall distribution.

**Table S3. Fitting parameters of pseudo-second-order and pseudo-first-order models.**

| **Adsorbents /Condition** | **Pseudo-first-order** | | |
| --- | --- | --- | --- |
|  | *R*^2^ | *q_e_* | *k*_1_ |
|  |  | mg g^−1^ | h^−1^ |
| UiO-66-NH_2_ Dark | 0.9038 | 37.09 | 1.66 |
| OVs-Zr/Fe UiO-66-NH_2_ Dark | 0.9782 | 139.55 | 1.72 |
| UiO-66-NH_2_ Light | 0.9907 | 299.6 | 0.37 |
| OVs-Zr/Fe UiO-66-NH_2_ Light | 0.9943 | 377.08 | 0.66 |

**Table S4. Formulation of simulated multication-containing solution.**

| **Metal ions** | **Concentration (ppm)** | **Relative molecular mass** | **Counterions** |
| --- | --- | --- | --- |
| U | 18.90 | 238 | NO_3_^2−^ |
| Ba^2+^ | 6.18 | 137.327 | NO_3_^2−^ |
| Cd^2+^ | 6.21 | 112.411 | NO_3_^2−^ |
| Co^2+^ | 3.08 | 58.93 | NO_3_^2−^ |
| Cu^2+^ | 4.18 | 63.55 | NO_3_^2−^ |
| Mg^2+^ | 1.53 | 24.31 | NO_3_^2−^ |
| Ni^2+^ | 3.30 | 58.69 | NO_3_^2−^ |
| Pb^2+^ | 10.10 | 207.2 | NO_3_^2−^ |
| Sr^2+^ | 4.22 | 87.62 | NO_3_^2−^ |
| Zn^2+^ | 3.34 | 65.41 | NO_3_^2−^ |

**Table S5. Details of the calculation of the *K*_d_ value.**

| **Adsorbents /Condition** | ***C_0_* (mg L^−1^)** | ***C_e_* (mg L^−1^)** | ***V* (mL)** | ***m* (g)** | ***K*_d_ (mg L^−1^)** |
| --- | --- | --- | --- | --- | --- |
| UiO-66-NH_2_ Dark | 18.90 | 16.18 | 100 | 0.005 | 3.36 🞨 10^3^ |
| OVs-Zr/Fe UiO-66-NH_2_ Dark | 18.90 | 13.53 | 100 | 0.005 | 7.93 🞨 10^3^ |
| UiO-66-NH_2_ Light | 18.90 | 10.34 | 100 | 0.005 | 1.65 🞨 10^4^ |
| OVs-Zr/Fe UiO-66-NH_2_ Light | 18.90 | 1.75 | 100 | 0.005 | 1.96 🞨 10^5^ |

**Table S6. Uranium tailings wastewater. Ion content analysis.**

| **Coexisting ions** | **Concentration**  **(ppm)** | **Coexisting ions** | **Concentration**  **(ppm)** |
| --- | --- | --- | --- |
| U | 0.5 | Fe^3+^ | 4.00×10^−2^ |
| Mn^2+^ | 12.0 | Cl^−^ | 1.09×10^2^ |
| Zn^2+^ | 6.9 | NO_3_^−^ | 3.85×10^2^ |
| Ca^2+^ | 4.18×10^2^ | NH^4+^-N | 9.85 |
| Mg^2+^ | 44.7 | COD | 35.0 |
| Cd^2+^ | 6.21 |  |  |

**Table S7. The generation of H_2_O_2_ under the light.**

| **Sample** | **1h** | **2h** |
| --- | --- | --- |
| UiO-66-NH_2_ | 0 | 0.81 μmol/g/h |
| OVs-Zr/Fe UiO-66-NH_2_ | 4.83 μmol/g/h | 3.22 μmol/g/h |
